# Diagnosis of foetal vein of galen aneurysmal malformation by ultrasound combined with magnetic resonance imaging: a case series

**DOI:** 10.1186/s12880-020-00463-6

**Published:** 2020-06-12

**Authors:** Tian-gang Li, Yao-yue Zhang, Fang Nie, Mei-juan Peng, Yun-Zhi Li, Pei-long Li

**Affiliations:** 1 Department of Ultrasound Diagnosis, Gansu Provincial Maternity and Child-care Hospital, Lanzhou, 730050 Gansu Province P. R. China; 2 Department of Ultrasound diagnosis, Lanzhou University Second Hospital, Lanzhou, 730030 Gansu Province P. R. China

**Keywords:** Ultrasound, magnetic resonance imaging, Foetus, vein of Galen aneurysmal malformation, Prenatal diagnosis, Congenital malformation

## Abstract

**Background:**

Foetal vein of Galen aneurysmal malformation (VGAM) is a very rare congenital malformation of the cerebral blood vessels. We sought to evaluate the diagnostic value of ultrasound in combination with magnetic resonance imaging (MRI) in foetal VGAM.

**Case presentation:**

Prenatal ultrasound combined with MRI diagnosed five cases of VGAM. Two dimensional ultrasound images were used to find the echo-free cystic structure below the thalamus and above the cerebellum with five cases. Colour blood flow showed dilated VGAM in five cases, while the arteriovenous spectrum was explored in two cases and foetal heart failure was found in other three cases. MRI was manifested as a dilated VGAM found at the midline of the brain, demonstrating widening or dilation of the straight sinus in four cases, ventricular dilatation in one case, brain parenchyma bleeding in two cases, and grey matter softening in one case. One infant died on the day of its birth, while the other four infants died within one month to six months after birth.

**Conclusions:**

Ultrasound combined with MRI can more accurately and comprehensively observe the pathological characteristics of VGAM, diagnose related complications early and determine its prognosis.

## Background

Foetal vein of Galen aneurysmal malformation (VGAM) is a very rare congenital malformation of the cerebral blood vessels, accounting for about 1% of foetal intracranial malformations [1]. These malformations form between the sixth and 11th week of gestation. Due to the fistula between the cerebral arteries and deep draining veins of the brain, the median prosencephalic vein, a precursor to the vein of Galen, becomes significantly enlarged and aneurysmal [2–4]. The condition is mostly detected during late pregnancy in the third trimester, often combined with foetal brain injury and cardiac dysfunction, and the prognosis is poor [5]. Earlier diagnoses have also been reported [6, 7].

VGAM leads to a typical ultrasound sonogram [1, 3]. Magnetic resonance imaging (MRI) can show the development of the intracranial nervous system and brain parenchymal damage [8]. With a study of previous literatures, ultrasound or MRI examination was used to diagnose foetal VGAM. There were rarely reports of analysis and diagnosis of VGAM by ultrasound combined MRI. This study analysed the imaging characteristics and value in diagnosing VGAM by prenatal ultrasound and MRI among five foetuses at Gansu Maternal and Child Health Hospital.

## Case presentation

### Characteristics and basic clinical information

Between January 2013 and December 2019, five cases of foetal VGAM were diagnosed by prenatal ultrasound and MRI examinations at Gansu Maternal and Child Health Hospital. Four cases were single infants and one case was one of the twins, and the age, parity, delivery method, ultrasound diagnosis week, gestational age, presence of heart failure, and clinical outcomes were evaluated (Table 1). We calculated the gestational age based on the first day of the last menstrual period and confirmed it by crown–rump length measurement on the first trimester-ultrasound. All cases had undergone at least one detailed ultrasound scan (two-dimensional and, more recently, three-dimensional) and as well as one MRI prenatally.

**Table 1:** Characteristics and basic clinical information of the five cases of VGAM

Case number	Maternal age	GA at diagnosis (weeks)	GA at delivery (weeks)	Birth weight (grams)	Number of infants	Parity	Delivery method
1	23y	34^+ 5^	35^+ 2^	2350	Twins	Primipara	Caesarean section
2	30y	30^+ 1^	39^+ 6^	2950	Single	Multiple	Natural delivery
3	36y	35^+ 6^	36^+ 1^	2300	Single	Multiple	Natural delivery
4	28y	40^+ 1^	40^+ 6^	3740	Single	Multiple	Natural delivery
5	23y	33^+ 3^	33^+ 5^	2180	Single	Primipara	Natural delivery

*GA* gestational age, *VGAM* vein of Galen aneurysmal malformation

### Clinical findings

**Fig. 1:**
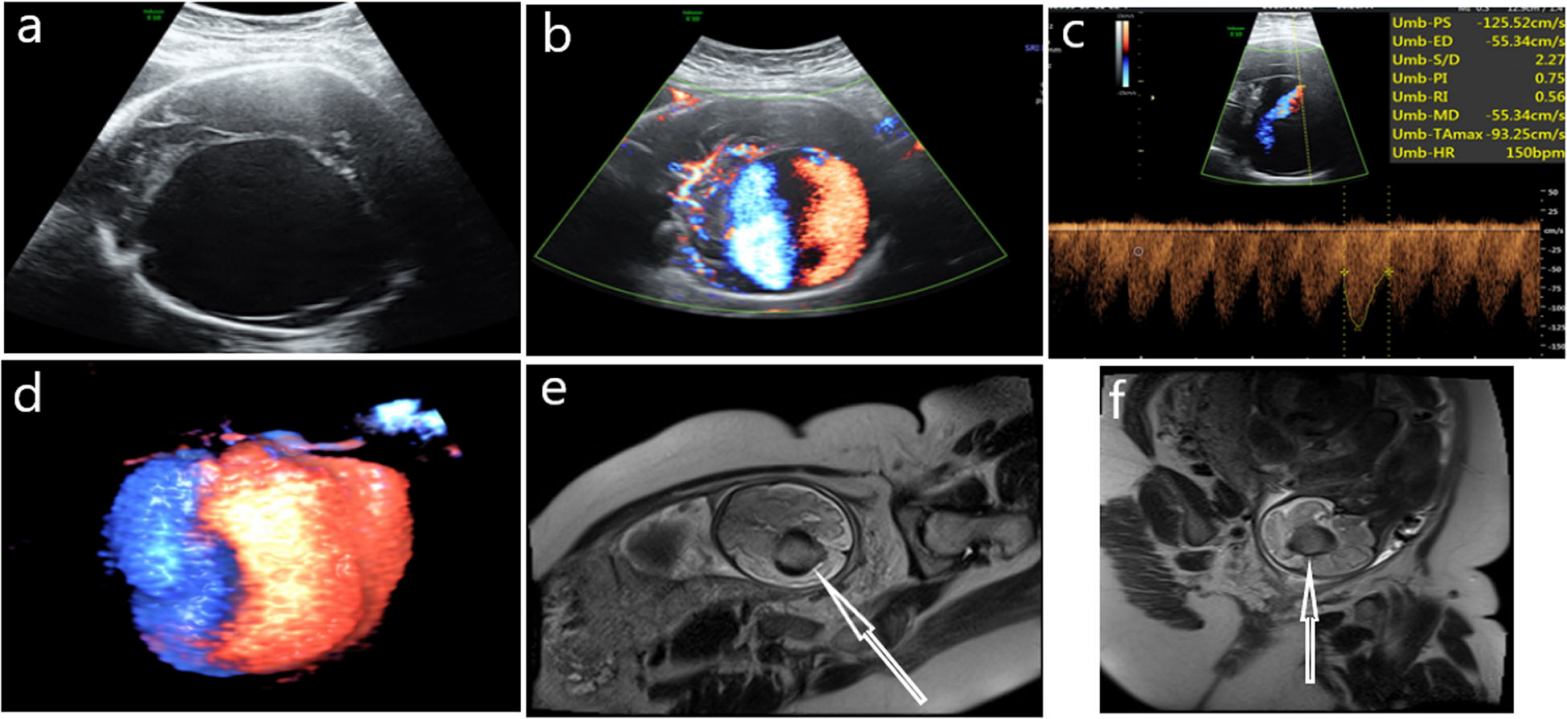
Case 1, 35-week prenatal ultrasound and MRI images of a VGAM foetus. **a**: Two-dimensional ultrasound shows a huge thin-walled echoless area next to the midline of the brain. **b**: Colour Doppler shows a full blood-flow signal in the brain’s echoless area. **c**: The skull’s echoless area shows a high-speed low-impedance blood-flow spectrum. **d**: The reconstructed three-dimensional energy Doppler image clearly shows the dilated Galen vein, but no obvious straight sinus is displayed. **e**: MRI cross-section of the foetal skull showing the dilated Galen vein through flow phenomenon (white arrow). **f**: Sagittal section of the foetal brain showing dilated Galen’s venous aneurysm by flow phenomenon (white arrow)

**Fig. 2:**
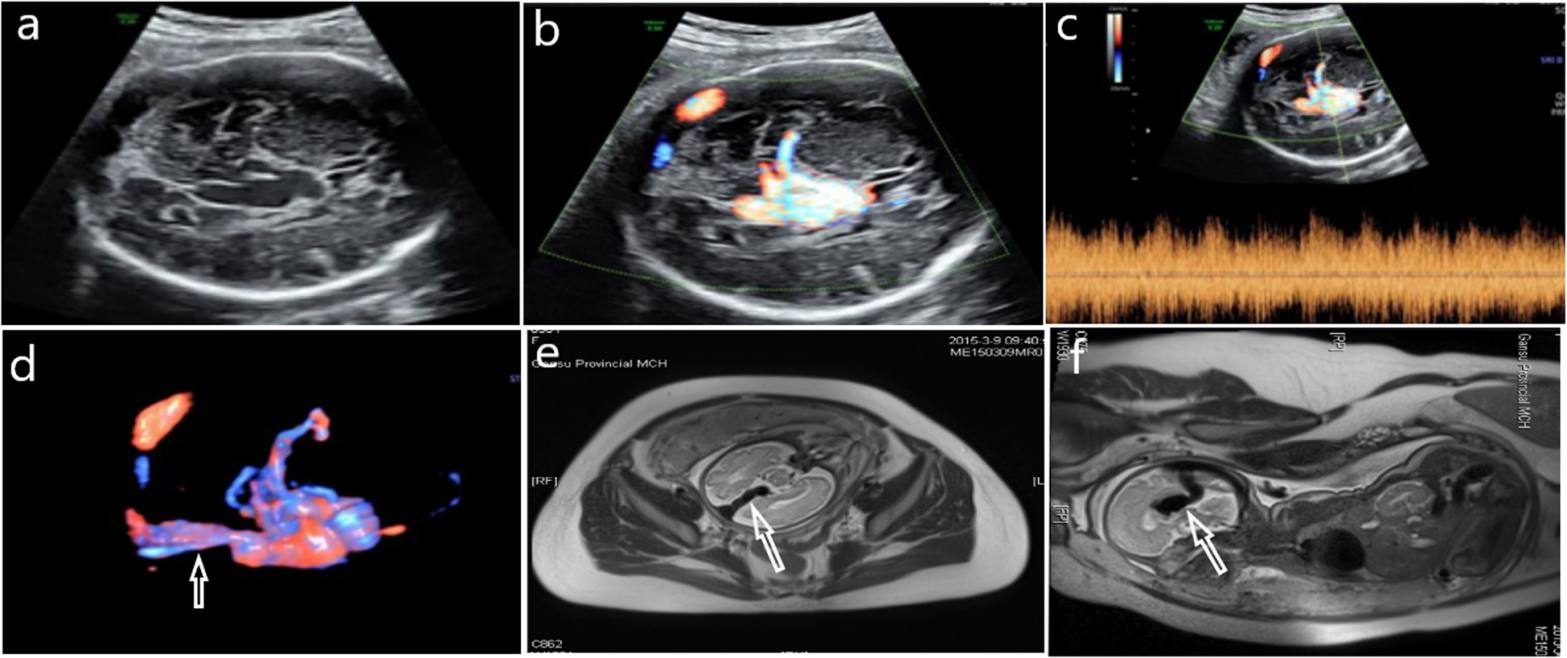
Case 2, 30-week prenatal ultrasound and MRI images of a VGAM foetus. **a**: Two-dimensional ultrasound shows a huge thin-walled echoless area next to the midline of the brain. **b**: Colour Doppler shows a full blood-flow signal in the brain’s echoless area. **c**: The skull’s echoless area shows arteriovenous leak with arteriovenous mixed spectrum; **d**: The reconstructed three-dimensional energy Doppler image clearly shows the dilated VGAM, and obvious straight sinus is displayed (white arrow). **e**: MRI cross-section of the foetal skull showing the dilated VGAM through flow phenomenon (shown by white arrow). **f**: Sagittal section of the foetal brain showing the dilated Galen’s venous aneurysm by flow phenomenon (white arrow)

In all five cases of foetal ultrasound, an echo-free cystic structure was found below the thalamus and above the cerebellum of approximately the same size across all cases and with clear boundaries (Figs. 1a and 2a), revealing that the blood flow in the nonechoic area was full (Figs. 1b and 2b). 3D blood flow imaging revealed the three-dimensional distribution of whole blood flow and blood flow in VGAM (Figs. 1c and 2c). Meanwhile, the spectrum approach explored the arteriovenous fistula and high-speed low resistance (Fig. 1d) or arteriovenous mixed spectrum (Fig. 2d), and MRI examination of the foetal head cross-section (Figs. 1e and 2e) revealed that the VGAM and the sagittal section (Figs. 1f and 2f) showed the VGAM straight sinus.

One case had a gestational age of less than 32 weeks at the time of ultrasound, while the other four cases had a gestational age of more than 32 weeks. Five foetuses were born, of which one was one of the twin pregnancy and four were single pregnancies. Three infants were premature, and all of them were SGA foetuses, with other basic information such as parity and delivery methods being described in Table 1.

The volume of VGAM could be measured by ultrasound and MRI and the results were similar. The maximum volume as measured by MRI was 150.12 cm^3^ and the minimum volume was 1.64 cm^3^. Three cases showed straight sinus dilatations with an inner diameter of more than 0.6 cm. All five cases had high-risk factors or comorbidities, including three that were accompanied by structural abnormalities of the foetal heart. After labour, one infant died on the day of its birth, while the other four cases died within one month to six months after birth (Table 2).

**Table 2:** Characteristics of the five cases of VGAM showing progression of the lesion from diagnosis to delivery

Case number	Volume of US (cm^3^)	Volume of MRI (cm^3^)	Straights dilatation (MRI)	High-risk factors or combined disease	CHD with foetus	Outcome
1	166.53	150.12	0.1	IVF	LSVC	Death
2	2.92	2.36	0.7	Hypothyroidism	VSD	Death
3	1.58	1.64	0.6	PIH	No	Death
4	13.53	12.36	0.4	Gestational diabetes	VSD	Death
5	9.84	10.15	1.1	PROM	No	Death

*US* ultrasound, *MRI* magnetic resonance image, *LSVC* left superior vena cava, *CHD* congenital heart disease, *PIH* pregnancy-induced hypertension, *IVF* in vitro fertilisation, *PROM* Premature membrane rupture, *VSD* ventricular septal defect

Ultrasound results showed arteriovenous leaks in two cases, foetal heart failure in three cases, and MRI findings in four cases suggesting widening or dilation of the straight sinus. One infant had ventricular dilatation and two had brain parenchyma bleeding. One case showed grey matter softening (Table 3).

**Table 3:** Characteristics of the five cases of VGAM showing diagnosis with US and MRI

Case number	Arteriovenous leak (US)	Heart failure (US)	Straight dilatation (MRI)	Ventricular dilatation (MRI)	Intraventricular haemorrhage (MRI)	White matter softening (MRI)
1	No	No	No	Yes	Yes	Yes
2	Yes	Yes	Yes	No	No	No
3	No	Yes	Yes	No	Yes	No
4	No	No	Yes	No	No	No
5	Yes	Yes	Yes	No	No	No

*US* ultrasound, *MRI* magnetic resonance image

Ultrasound was used to evaluate heart failure in five cases, with three subsequently diagnosed. The cardiothoracic ratio increased in three cases, one case showed pleural effusion, one case demonstrated peritoneal effusion, three cases showed tricuspid regurgitation, and three cases presented an abnormal umbilical artery spectrum. The frequency spectrum of the umbilical vein was abnormal in one case and that of the uterine artery was abnormal in one case (Table 4).

**Table 4:** Evaluation index and method of foetal cardiac function in five cases of VGAM with US

Case number	CTR	Pleural effusion (US)	Peritoneal effusion	TR	UA spectrum	UV spectrum	Uterine artery spectrum
1	Normal	No	No	No	Normal	Normal	Normal
2	Increase	Yes	No	Yes	Abnormal	Normal	Normal
3	Increase	No	Yes	Yes	Abnormal	Abnormal	Abnormal
4	Normal	No	No	No	Normal	Normal	Normal
5	Increase	No	No	Yes	Abnormal	Normal	Normal

*CTR* cardiothoracic ratio, *TR* tricuspid regurgitation, *UA* umbilical artery, *UV* umbilical vein

### Ultrasonography

We used E10 or E8 (GE Healthcare, USA) ultrasound systems with obstetric preset and completed foetal biometric measurements on each scan. Subsequently, we obtained a two-dimensional image of the VGAM from each patient to observe its shape and size. In particular, the following parameters were recorded: orthogonal diameters of the VGAM (i.e., craniocaudal, laterolateral, anteroposterior); volume of the VGAM, calculated from the three diameters with the ellipsoid formula; and presence/absence of straight sinus dilatation. Colour Doppler blood flow was used to detect blood-flow filling in VGAM and to observe the distribution of cerebral arteries and VGAM connection vessels, while the spectrum was used to monitor the blood flow spectrum. Finally, we observed whether the echo of the brain tissue surrounding the VGAM was enhanced and/or whether small cystic lesions were visible and determined whether there were softening lesions in the white matter of the foetus; further, real-time three-dimensional power Doppler sonography was used to observe the relations between the VGAM and cerebral artery branch and that between the VGAM and sagittal sinus connection, respectively. In addition, ultrasound assessment included foetal echocardiography, which sought to assess cardiac overload: after ruling out major cardiac anomalies, the cardiothoracic ratio (CTR), tricuspid regurgitation, umbilical artery spectrum and middle cerebral artery spectrum were evaluated. The CTR, measured as the cardiac area/thoracic area on a four-chamber axial view of the foetal thorax, was considered abnormal if greater than 0.50 [9]. Tricuspid regurgitation was considered present and, thus, abnormal, if holosystolic and/or with a maximum velocity of more than 2 m/sec [10]. Umbilical artery, umbilical vein and uterine artery spectrum measurements were conducted to determine the basic situation of the foetus.

### MRI examination

Prenatal MRI was performed using a 1.5-Tesla MRI system (Siemens, Munich, Germany) with the HASTE fast imaging sequence, single-shot fast-spin echo, and true fast imaging with steady-state precession (True FISP) to gather scans of the transverse, coronal and sagittal planes of the foetal brain.

The VGAM volume in cm^3^ was calculated—both on US and MRI—with the ellipsoid formula [v = 4/3*3.14* (abc), with a, b and c constituting the three orthogonal diameters, respectively] [8].

## Discussion and Conclusions

The prenatal diagnosis of VGAM is usually made during the third trimester. Colour Doppler ultrasonography is most often used as the modality for exploring the foetus [11], demonstrating turbulent arterial and venous flow within a hypoechogenic structure located in the midline of the posterior part of the third ventricle [4, 11, 12]. However, foetal MRI has become superior to colour Doppler ultrasonography in the diagnosis of VGAM in recent years [13]. With the application of MRI in the foetus, VGAM can be more intuitively diagnosed and the situation of adjacent anatomy can be analysed, including the blood and cerebral ischaemic areas. In addition, MRI has high specificity and sensitivity in diagnosing brain VGAM and can accurately display the size and location of the VGAM and its relationship with adjacent brain tissue [14], so applying MRI in the diagnosis of VGAM is a valuable technique. The limitation of foetal MRI is that it cannot evaluate the blood-flow spectrum of arteriovenous fistula or foetal cardiac function, and the latter is closely related to prognosis [15]. The five cases in this study were first discovered during a routine ultrasound examination that clearly showed the internal blood flow of VGAM and the presence of arteriovenous leaks, indicating that ultrasound achieves a better sensitivity in detecting VGAM than MRI, with three-dimensional ultrasound being able to directly display the overall shapes of VGAM and the expansion of the straight sinus. In this study, all cases were found in the third trimester, which was consistent with the details of prior literature reports [11, 12].

The value of MRI and ultrasound in predicting the outcome of antenatally diagnosed VGAM has rarely been reported [16]. The cerebral shunt created by the aneurysm can increase the cardiac preload and lead to congestive heart failure. Intrauterine ultrasound signs of heart failure, such as cardiomegaly, tricuspid insufficiency, polyhydramnios, pericardial and pleural effusion, oedema and ascites carry a poor prognosis and indicate an intractable high-flow anomaly [17]. Among the three cases of heart failure diagnosed in this study, the heart function was assessed in detail by two-dimensional and colour Doppler through ultrasound. The authors thus believe that ultrasound has a clear advantage over MRI in evaluating foetal heart function.

The prognosis of VGAM depends upon two factors. First is the severity of heart failure, which is directly related to the number of arteriovenous shunts. This study shows that ultrasound can objectively reflect whether VGAM foetuses have heart failure and the degree of severity of the heart failure. In this study, smaller tumours are more prone to accompanying heart failure, while larger tumours are less likely to be paired with such. The main reason for this is that the straight sinus is more dilated when the tumour is smaller and, thus, most of the blood flow in the tumour passes through the tumour into the straight sinus. Important in this discussion, heart failure is caused by increased blood flow to the heart. When the tumour is large, arteriovenous leakage persists, the tumour expands and the straight sinus is not dilated. The blood flow in larger tumours does not flow well into the straight sinus, so such rarely causes heart failure. In this study, two cases of cardiac insufficiency were found by ultrasound, both of which showed an increased cardiothoracic ratio and straight sinus dilatations that were more obvious; another factor that determines the prognosis of VGAM is an increase in the internal venous pressure of VGAM, which causes the brain tissue surrounding VGAM to experience ischaemic changes. This study supports that MRI can clearly reveal changes in the brain tissue around the VGAM, which has advantages over ultrasound examination. This study further indicates that foetal heart failure is likely to be associated with smaller tumours, and changes in the brain tissue surrounding the foetal VGAM are likely to occur when the tumour is larger, both of which are responsible for the poor overall prognosis of the VGAM foetus. In summary, the authors believe that, when VGAM is suspected before birth, both ultrasound and MRI should be used for examination and evaluation to optimally benefit the overall prognosis of the foetus.

Previously, Deloison et al. [18] found that the prenatal diagnosis of VGAM with other abnormal foetal conditions has a poor prognosis, while isolated foetal VGAM often exhibits a better prognosis. In addition, because VGAM is typically found in the third trimester, if routine ultrasound examinations are not performed during the third trimester of pregnancy, cardiac insufficiency symptoms are often found only after the foetus is born. Therefore, when unexplained cardiac insufficiency occurs after birth, a detailed inspection should be conducted to determine whether this disease is at play. In this study, although all of the five cases continued to progress to birth, all infants died in the neonatal period after birth, which was consistent with the poor prognosis of the condition reported in the literature [19].

Recent studies have found that VGAM is often associated with aortic transition position, ventricular septal defect, atrioventricular septal defect and other cardiac structural abnormalities [18]. In this study, two cases presented with ventricular septal defect and one case demonstrated left superior vena cava, consistent with other findings in literature reports [19]. Interestingly, all of the five pregnant women involved in this study had high-risk factors or comorbidities, including in vitro fertilisation and premature membrane rupture in one each and complications during pregnancy, including hypertension during pregnancy, decreased thyroid function, and gestational diabetes, in the other three, suggesting that foetuses exposed to maternal high-risk factors or comorbidities are more likely to develop VGAM.

An advantage of this investigation is that the collected prenatal data include detailed foetal neurosonographic, echocardiographic and MRI measurements in all cases. Regarding the limitations of our study, the small size of the foetal population is of concern but attributed the rarity of VGAM and the fact that the condition is usually diagnosed postnatally. Second, as all infants in this study died during the neonatal period, no long-term clinical treatment or treatment effect evaluation could be performed.

Prenatal ultrasound is very important in the diagnosis of VGAM. When foetal intracranial midline cystic lesions are found, colour Doppler and pulse Doppler can be used to observe the nature, direction, and spectrum of blood flow and to distinguish arteries and veins while carefully observing indicators closely related with the prognosis of the foetus such as cardiac function status and damage to the nervous system. Next, three-dimensional ultrasound can be used to image the vascular structure, elucidating the relationship between the supplying artery and the dilated Galen vein; Finally, MRI may be applied to further judge the nerves’ phylogeny and extent of damage. The combined application of prenatal ultrasound and MRI can accurately diagnose VGAM and related complications early and provide a reference to the evaluation of foetal prognosis. Thus, we believe that three-dimensional colour Doppler rendering may be used effectively to assess the general architecture of the lesion and the straight sinus size, but volume calculation should be conducted on the basis of three-dimensional ultrasound and MRI. Based on this study, ultrasound combined with MRI can more accurately and comprehensively observe the pathological characteristics of VGAM, diagnose related complications early and determine its prognosis.

## Data Availability

The data and material in the current study are available from the corresponding author on reasonable request.

## Abbreviations

VGAM: Vein of Galen aneurysmal malformation
MRI: Magnetic resonance imaging
CTR: Cardiothoracic ratio
GA: Gestational age
US: Ultrasound
LSVC: Left superior vena cava
CHD: Congenital heart disease
PIH: Pregnancy-induced hypertension
IVF: in vitro fertilisation
PROM: Premature membrane rupture
VSD: Ventricular septal defect
TR: Tricuspid regurgitation
UA: Umbilical artery
UV: Umbilical vein
